# Quantitative anatomical analysis of lumbar interspaces based on 3D CT imaging: optimized segment selection for lumbar puncture in different age groups

**DOI:** 10.1007/s00234-023-03272-0

**Published:** 2024-01-06

**Authors:** Yuan-Dong Zhuang, Xiao-Cong Hu, Ke-Xin Dai, Jun Ye, Chen-Hui Zhang, Wen-Xuan Zhuo, Jian-Feng Wu, Shi-Chao Liu, Ze-Yan Liang, Chun-Mei Chen

**Affiliations:** 1 Department of Neurosurgery, Fujian Medical University Union Hospital, Fujian Institute of Neurosurgery, No. 29 Xinquan Rd, Gulou District, Fuzhou, 350001 Fujian China; 2 Fujian Medical University, No. 1 Xuefu North Rd, Minhou County, Fuzhou, 350100 Fujian China

**Keywords:** Lumbar puncture, Segment selection, 3D modeling, Efficacy ratio, Age factor

## Abstract

**Background:**

Optimal lumbar puncture segment selection remains controversial. This study aims to analyze anatomical differences among L3-4, L4-5, and L5-S1 segments across age groups and provide quantitative evidence for optimized selection.

**Methods:**

80 cases of CT images were collected with patients aged 10–80 years old. Threedimensional models containing L3-S1 vertebrae, dural sac, and nerve roots were reconstructed. Computer simulation determined the optimal puncture angles for the L3-4, L4-5, and L5-S1 segments. The effective dural sac area (ALDS), traversing nerve root area (ATNR), and area of the lumbar inter-laminar space (ALILS) were measured. Puncture efficacy ratio (ALDS/ALILS) and nerve injury risk ratio (ATNR/ALILS) were calculated. Cases were divided into four groups: A (10–20 years), B (21–40 years), C (41–60 years), and D (61–80 years). Statistical analysis was performed using SPSS.

**Results:**

1) ALDS was similar among segments; 2) ATNR was greatest at L5-S1; 3) ALILS was greatest at L5-S1; 4) Puncture efficacy ratio was highest at L3-4 and lowest at L5-S1; 5) Nerve injury risk was highest at L5-S1. In group D, L5-S1 ALDS was larger than L3-4 and L4-5. ALDS decreased after age 40. Age variations were minimal across parameters.

**Conclusion:**

The comprehensive analysis demonstrated L3-4 as the optimal first-choice segment for ages 10–60 years, conferring maximal efficacy and safety. L5-S1 can serve as an alternative option for ages 61–80 years when upper interspaces narrow. This study provides quantitative imaging evidence supporting age-specific, optimized lumbar puncture segment selection.

## Introduction

Lumbar puncture (LP) is an essential diagnostic and therapeutic procedure for various neurological conditions [1]. However, the optimal lumbar segment selection remains controversial. Some studies have advocated L3-4 [2, 3], while others recommended L4-5 [4] or L5-S1 [5, 6]. Most recommendations lack quantitative evidence on the anatomical differences among segments.

Patient age may also impact the optimal lumbar puncture segment [7]. However, few studies have examined age-related anatomical changes that could inform optimal segment selection [8].

To address these knowledge gaps, this study aimed to quantify key lumbar anatomical parameters across different age groups using 3D modeling technology. This novel approach allowed the reconstruction of digital virtual human (DVH) models from CT images. We obtained measurements of the dural sac area, traversing nerve root area, and interlaminar window area at the L3-4, L4-5, and L5-S1 segments. Furthermore, we proposed the concepts of puncture efficacy ratio and nerve injury risk ratio to evaluate the advantages of different intervertebral spaces using imaging quantifications.

Age-related variations in quantitative anatomy were also analyzed, a perspective scarcely discussed before [9]. The findings may provide insights into anatomical changes with aging and among segments, enhancing the understanding of optimal patient-tailored LP techniques that balance safety and efficacy [10].

## Materials and methods

### Clinical data collection

We retrospectively enrolled patients aged 10–80 years who underwent lumbar CT scans (0.6 mm slice thickness) at our hospital (Fujian Medical University Union Hospital, China) from January to December 2019. Lumbar CT images containing at least L3 to S1 levels were randomly selected.

Inclusion criteria were age 10–80 years and body mass index (BMI) of 18.5–40 kg/m2 [11, 12]. Exclusion criteria were: 1) ankylosing spondylitis; 2) lumbar scoliosis with Cobb angle ≥ 20° [13]; 3) severe lumbar anatomical variations; 4) congenital spinal defects; 5) history of lumbar surgery.

Patients were categorized into 4 age groups: Group A (10–20 years, *n* = 20), Group B (21–40 years, *n* = 20), Group C (41–60 years, *n* = 20), and Group D (61–80 years, *n* = 20). CT images were exported in DICOM format for 3D reconstruction.

The study was approved by the Institutional Review Board. Written informed consent was obtained from all participants.

### Lumbar spine 3D model reconstruction

**Fig. 1:**
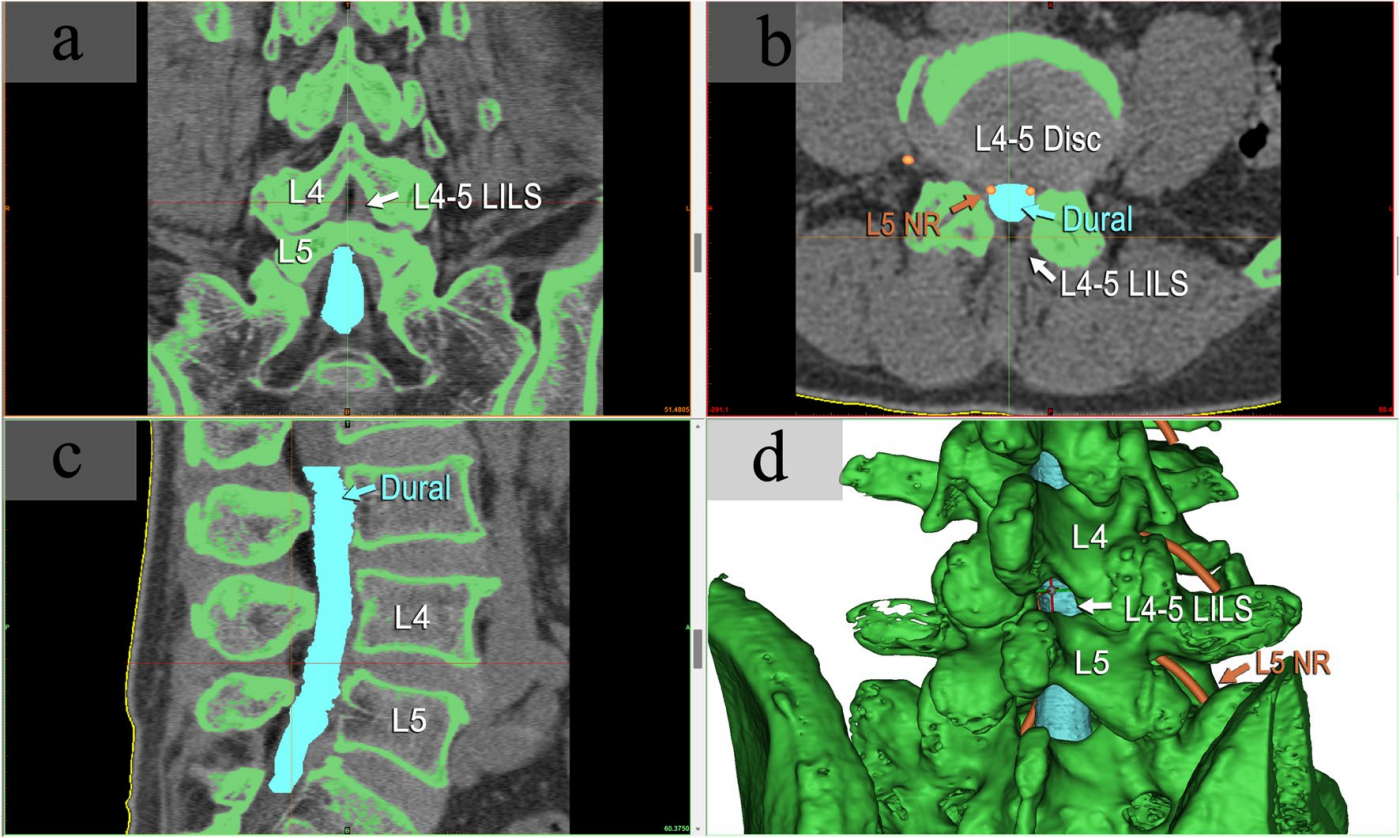
Three-dimensional reconstruction of lumbar models from CT images. Lumbar spine models containing the vertebral bone (green), dural sac (blue), and nerve roots (orange) were reconstructed from CT thin-slice images using Mimics software. The anatomy was delineated on coronal (**a**), transverse (**b**), and sagittal (**c**) planes by adjusting contrast and applying anatomical knowledge. This enabled precise 3D modeling (**d**) for subsequent measurement of key anatomical parameters related to lumbar puncture. Abbreviations: LILS—Lumbar interlaminar space, NR—Nerve root

The collected lumbar CT images (in DICOM format) containing at least L3-S1 levels were imported into Mimics Research 20.0 software (Materialise, Belgium). Based on CT grayscale and anatomical knowledge, target structures including vertebral bone, dura mater, nerve roots, and skin were delineated on transverse, sagittal, and coronal planes by adjusting the window width and level to enhance the region of interest (ROI) contrast. 3D reconstruction was performed to create distinct 3D models of the bone (green), dural sac (blue), and L3-S1 nerve roots (orange) (Fig. 1).

### Simulation of optimal needle insertion angle using conventional midline approach technique (MAT)

The conventional midline approach technique (MAT) for lumbar puncture was simulated using a virtual camera model. The needle insertion point and trajectory were modeled as the camera axis. The visible target area in the simulated camera view represented the permissible range for needle planning, where a larger area indicates greater error tolerance.

**Fig. 2:**
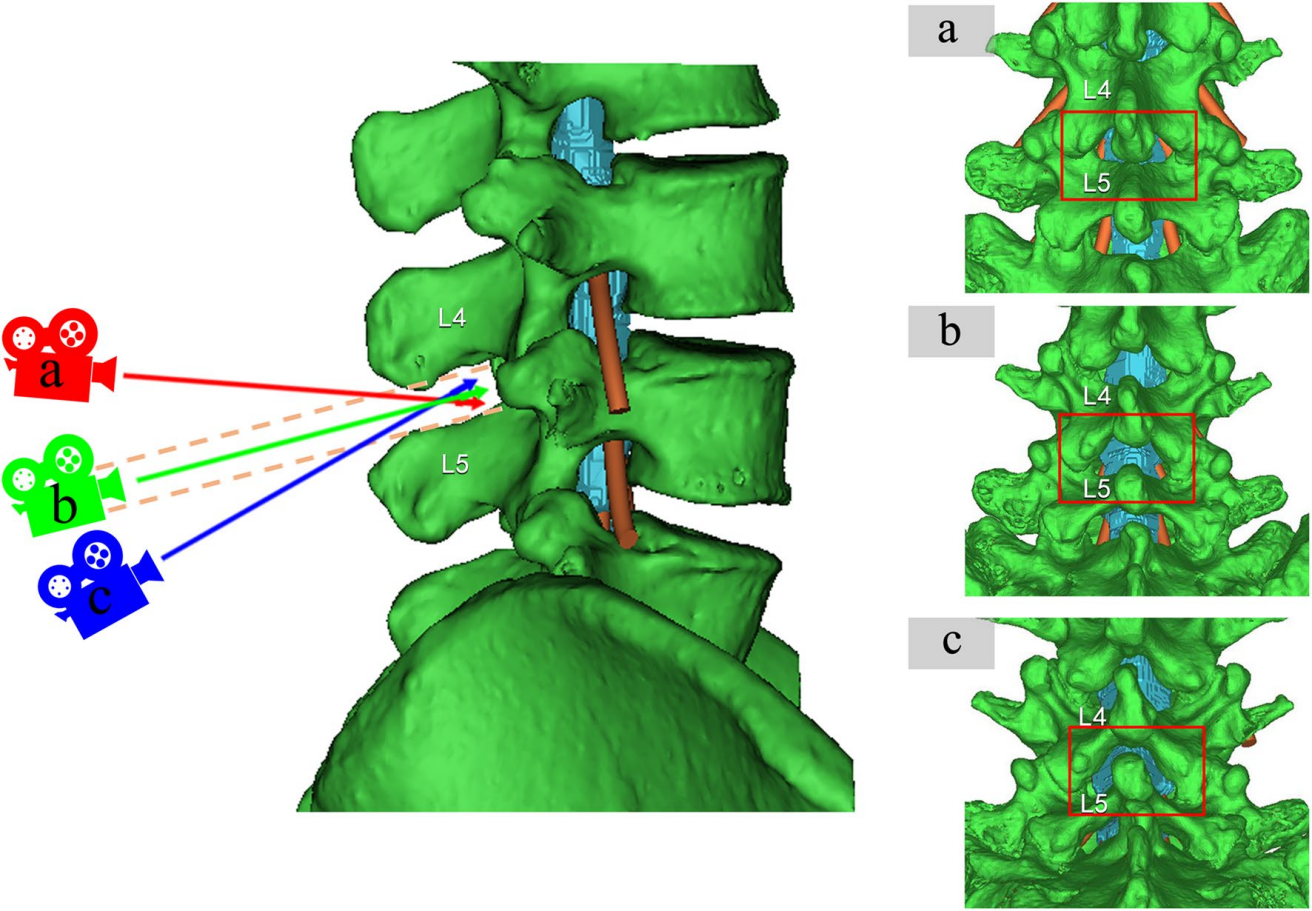
Simulation of optimal needle trajectory angle for midline approach lumbar puncture at L4-L5 segment as an example. The virtual camera axis simulated different needle trajectory angles along the midline. Larger visible dural sac area (blue) indicates greater potential to access that region during puncture. The optimal angle (**b**) had the camera axis parallel to the L4-L5 interspinous line (orange dotted line), maximizing visible interlaminar dural sac area. Excessive caudal tilting (**a**) or cranial tilting (**c**) led to smaller visible areas and were discarded. This process selected the optimal MAT trajectory angle for further analysis

The area of the lumbar dural sac (ALDS) within the interlaminar space represents the puncture target. Camera angles were screened in the mid-sagittal plane to maximize ALDS, implying higher error tolerance and success rate. Images were captured at the optimal angle for further analysis (Fig. 2). This process was repeated for the L3-4, L4-5, and L5-S1 segments.

### Measurement of ALDS, ATNR, and ALILS for respective puncture segments

**Fig. 3:**
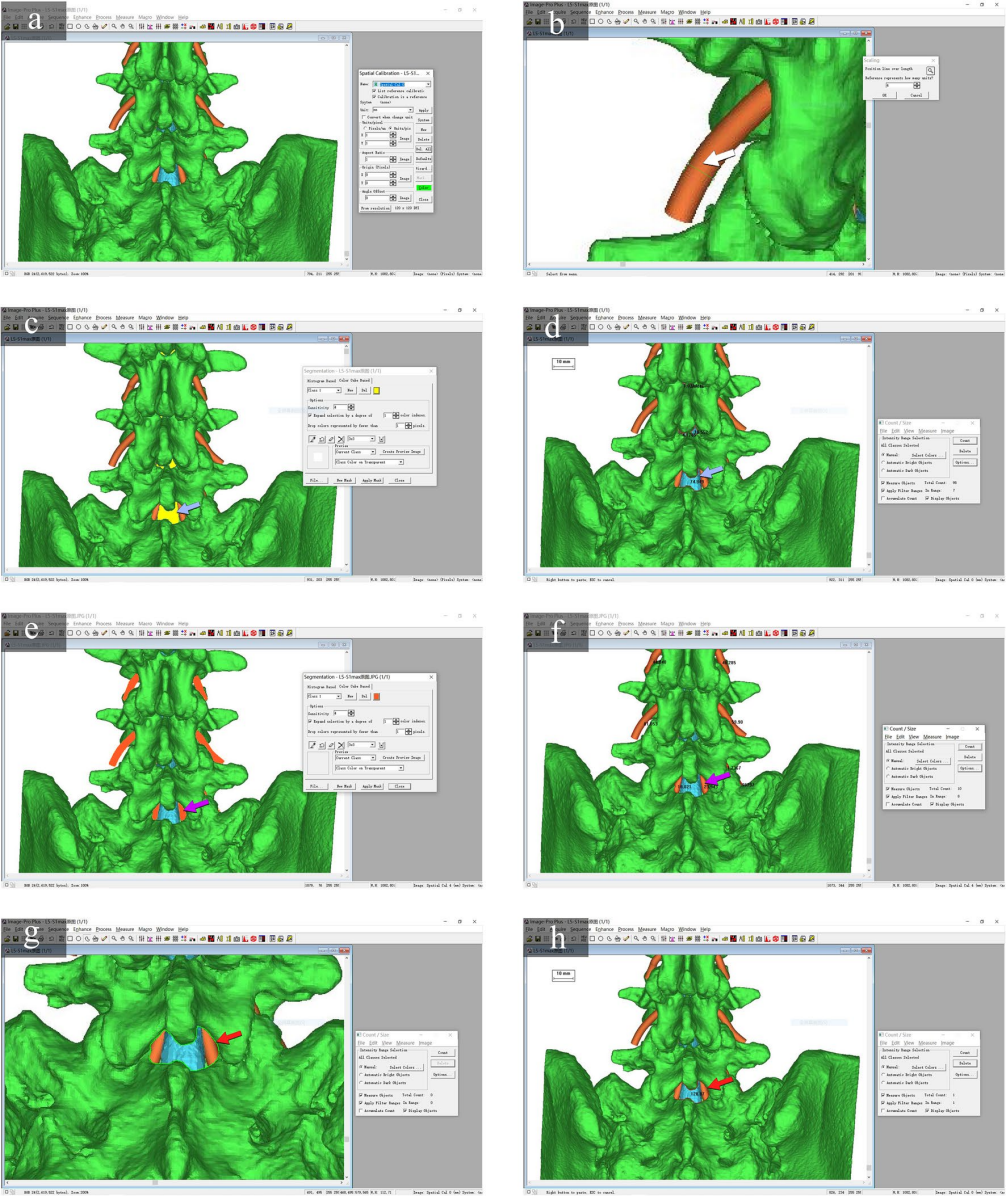
Measurement of ALDS, ATNR, and ALILS using IPP software with L5-S1 as an example. **a** The optimized simulated MAT puncture angle image obtained in the previous step was imported into IPP software for area analysis. **b** A scale bar was established based on the known diameter of anatomical structures like nerve roots (white arrow, 4 mm here). **c** The dural sac area (yellow shading) was delineated, blue arrow. **d** The dural sac area (ALDS) was measured, 74.9 mm^2^ here. **e** The traversing nerve root area (orange shading) was selected, purple arrow. **f** The nerve root area (ATNR) was measured, 42.0 mm^2^ here. **g** The interlaminar window (light green) was outlined, red arrow. **h** The lumbar interlaminar space area (ALILS) was calculated, 128.9 mm^2^ here

**Fig. 4:**
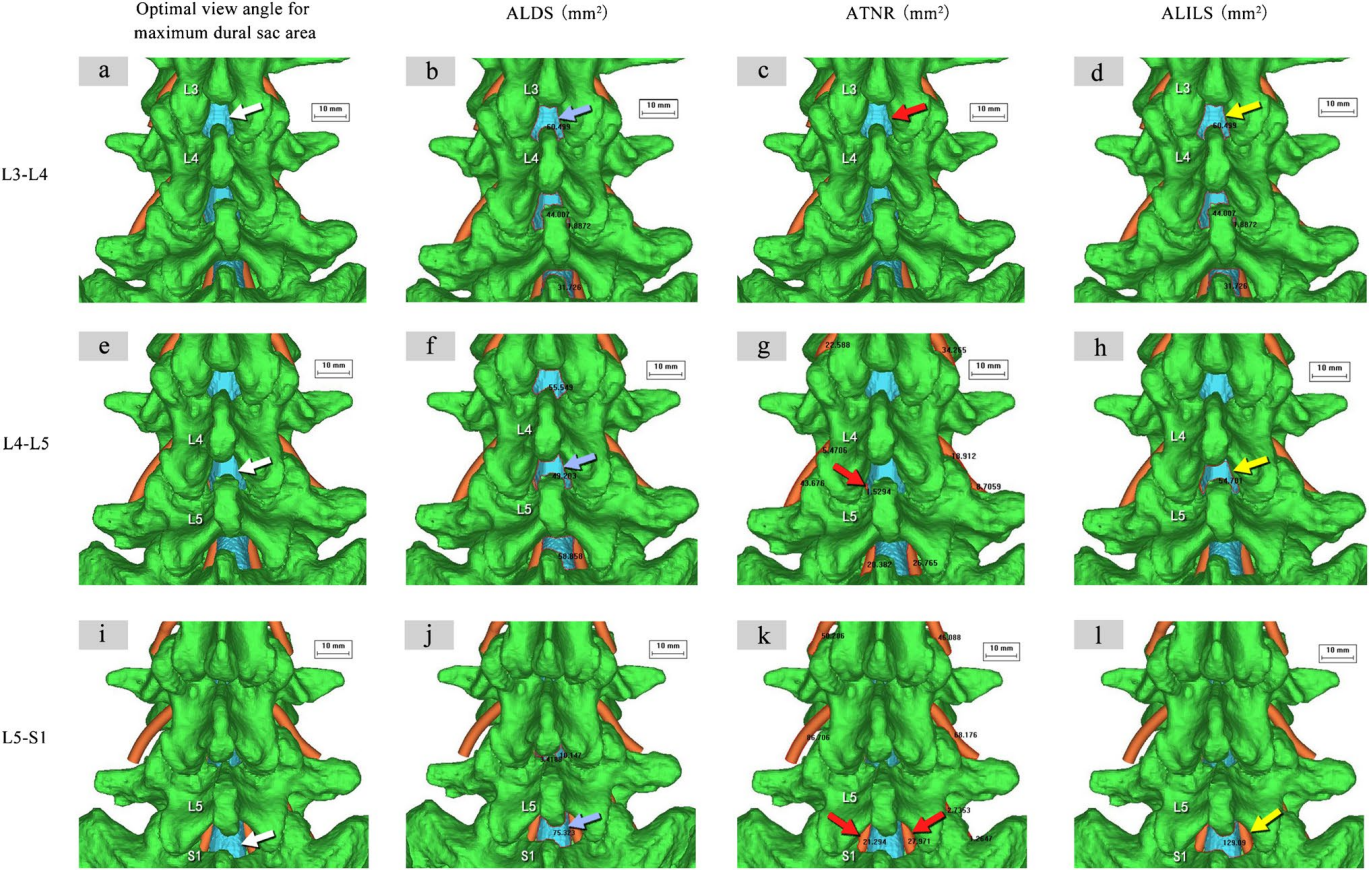
Measurement of ALDS, ATNR, and ALILS at optimized simulated puncture angles for L3-4, L4-5, and L5-S1. Optimal trajectory angles visualizing maximal dural sac area were determined for (**a**) L3-4, (**e**) L4-5, and (**i**) L5-S1. Key parameters were then measured on images captured at these angles: **b**, **f**, **j** dural sac area (ALDS, blue arrows), **c**, **g**, **k** traversing nerve root area (ATNR, red arrows), and (**d**, **h**, **l**) interlaminar window area (ALILS, yellow arrows). This enabled standardized comparison of the anatomical dimensions for the different segments

The captured images of the L3-4, L4-5, and L5-S1 segments at the optimized simulated puncture angles were imported into Image-Pro Plus 6.0 software (IPP, Media Cybernetics, USA). The following parameters were measured on each image: effective area of the lumbar dural sac (ALDS), area of the traversing nerve root (ATNR), and area of the lumbar interlaminar space (ALILS) (Figs. 3 and 4).

### Calculation of Puncture efficacy ratio (ALDS/ALILS)

The puncture efficacy ratio of the interlaminar window for each segment was calculated as Puncture efficacy ratio (%) = (ALDS/ALILS) × 100%. Where ALDS is the effective area of the lumbar dural sac, and ALILS is the area of the lumbar interlaminar window.

### Calculation of Nerve injury risk ratio (ATNR/ALILS)

The nerve injury risk ratio for each segment was calculated as Nerve injury risk ratio (%) = (ATNR/ALILS) × 100%. Where ATNR is the area of the traversing nerve root, and ALILS is the area of the lumbar interlaminar window.

### Statistical analysis

Statistical analysis was performed using SPSS version 23.0 (IBM, USA). The chi-square test was used for categorical variables. Continuous variables were expressed as mean ± standard deviation. An Independent t-test was used for normally distributed data, otherwise, non-parametric tests were applied. *P* < 0.05 was considered statistically significant.

ALDS, ALILS, ATNR, puncture efficacy ratio (ALDS/ALILS), and nerve injury risk ratio (ATNR/ALILS) were compared among the L3-4, L4-5, and L5-S1 segments. Subgroup analysis was also conducted across the four age groups.

## Result

### Baseline characteristics

The study included 80 patients, with 20 cases in each age group: 10–20 years, 21–40 years, 41–60 years, and 61–80 years. There was no significant difference in gender distribution among the groups (*P* > 0.05) (Table 1).

**Table 1:** Baseline characteristics (*n* = 80)

	Male	Female	*P*-value
Total (*n* = 80)	40	40	> 0.05
Group A: 10–20 yr (*n* = 20)	11	9	/
Group B: 21–40 yr (*n* = 20)	10	10	/
Group C: 41–60 yr (*n* = 20)	9	11	/
Group D: 61–80 yr (*n* = 20)	10	10	/
*P*-value	> 0.05	> 0.05	/

*P* < 0.05 indicates statistical significance

### Area of lumbar dural sac (ALDS) of puncture segments

In the overall age group, no significant differences in ALDS were observed among L3-4, L4-5, and L5-S1 segments (*P* > 0.05). However, in the 61–80 year age group, the ALDS of L5-S1 (69.4 ± 37.8 mm^2^) was markedly larger than L3-4 (43.6 ± 24.3 mm^2^) and L4-5 (41.6 ± 19.1 mm^2^) (*P* < 0.05).

**Fig. 5:**
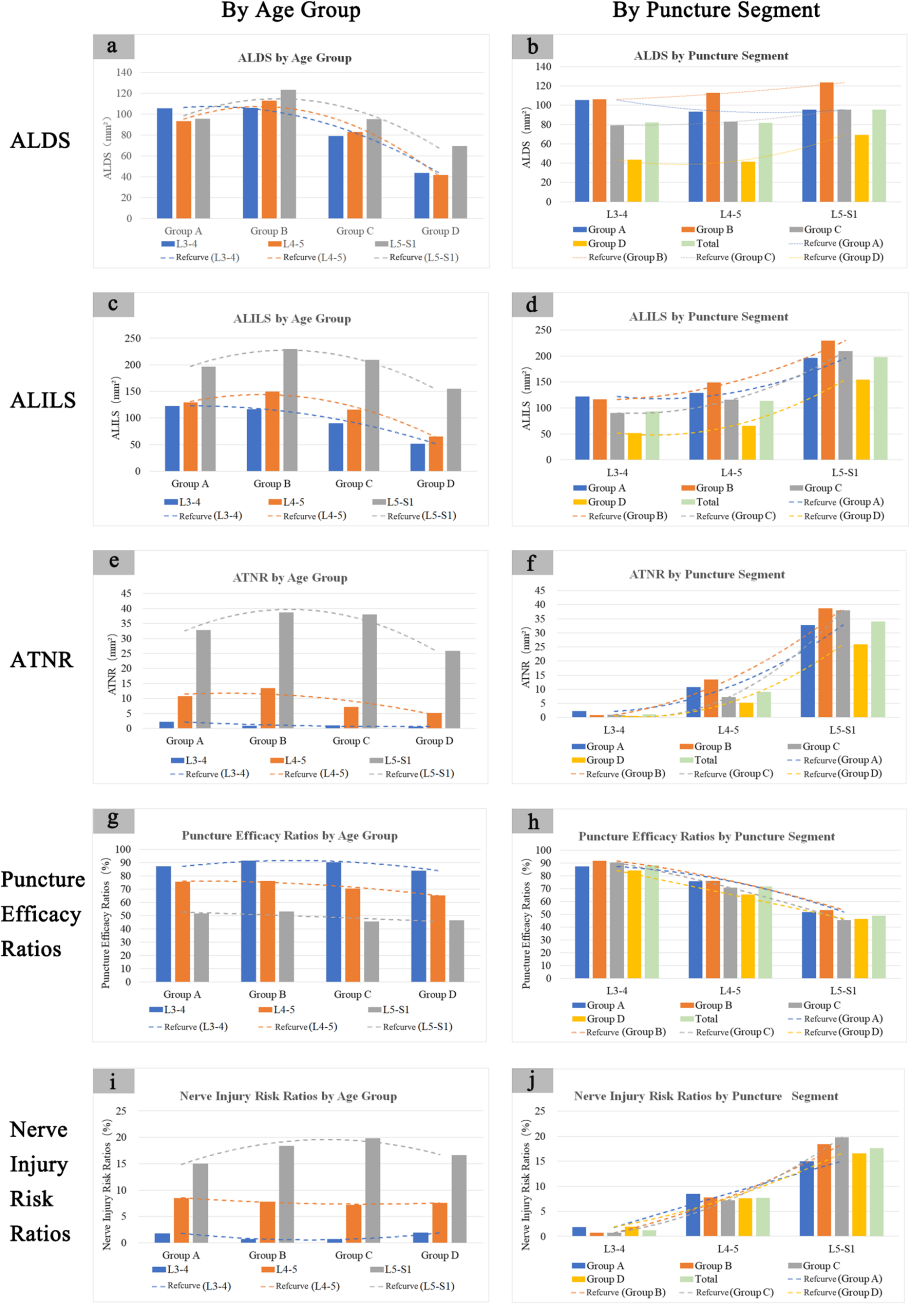
Histogram and trend lines depicting key lumbar anatomical measurements by age group and puncture segment. Dotted lines show measurement trends. Clustered columns display the 5 key indices (ALDS, ALILS, ATNR, Puncture Efficacy Ratios, Nerve Injury Risk Ratios) stratified by age group and segment. Panels (**a**, **b**) show ALDS. Panels (**c**, **d**) present ALILS. Panels (**e**, **f**) exhibit ATNR. Panels (**g**, **h**) visualize Puncture Efficacy Ratios. Panels (**i**, **j**) summarize Nerve Injury Risk Ratios. Different colored dotted lines indicate trends for corresponding measurements

A decreasing ALDS trend was noted after age 40 across segments. Within each segment, ALDS remained relatively stable between 10–40 years, followed by a gradual decrease. The decrease in ALDS occurred later and was smaller in magnitude at L5-S1 versus L3-4 and L4-5. In the overall study cohort, no significant ALDS differences existed among segments across the age range (*P* > 0.05) (Table 2 and Fig. 5).

**Table 2:** Key measurement indices across age groups and puncture segments

Indicators	Groups	L3-4	L4-5	L5-S1	*P*-value
ALDS (mm^2^)	Total (*n* = 80)	82.1 ± 45.7	81.7 ± 51.9	95.6 ± 54.0	*P* = *0.155* > *0.05*
	Group A: 10–20 yr (*n* = 20)	105.4 ± 49.8^ef^	93.3 ± 31.6^f^	95.5 ± 38.7	*P* = *0.655* > *0.05*
	Group B: 21–40 yr (*n* = 20)	106.0 ± 36.8^gh^	112.8 ± 44.9^gh^	123.4 ± 58.0^h^	*P* = *0.508* > *0.05*
	Group C: 41–60 yr (*n* = 20)	79.2 ± 41.6^egi^	82.8 ± 66.4^gi^	95.2 ± 54.8	*P* = *0.576* > *0.05*
	Group D: 61–80 yr (*n* = 20)	43.6 ± 24.3^bfhi^	41.6 ± 19.1^cfhi^	69.4 ± 37.8^bch^	*P* = *0.003* < *0.05*
	*P-value*	*P* < *0.001*	*P* < *0.001*	*P* = *0.008* < *0.05*	*/*
ATNR (mm^2^)	Total (*n* = 80)	1.1 ± 3.1^ab^	8.965 ± 14.0^ac^	34.0 ± 27.8^bc^	*P* < *0.01*
	Group A: 10–20 yr (*n* = 20)	2.2 ± 3.9^b^	10.8 ± 14.1^c^	32.8 ± 29.1^bc^	*P* < *0.01*
	Group B: 21–40 yr (*n* = 20)	0.9 ± 2.2^ab^	13.4 ± 20.0^ac^	38.9 ± 26.0^bc^	*P* < *0.01*
	Group C: 41–60 yr (*n* = 20)	1.0 ± 3.9^b^	7.2 ± 11.6^c^	38.0 ± 28.5^bc^	*P* < *0.01*
	Group D: 61–80 yr (*n* = 20)	0.6 ± 1.3^b^	5.2 ± 7.6^c^	25.9 ± 22.2^bc^	*P* < *0.01*
	*P-value*	*P* = *0.432* > *0.05*	*P* = *0.244* > *0.05*	*P* = *0.370* > *0.05*	*/*
ALILS (mm^2^)	Total (*n* = 80)	93.4 ± 51.0^b^	113.8 ± 65.3^c^	197.6 ± 90.3^bc^	*P* < *0.001*
	Group A: 10–20 yr (*n* = 20)	122.2 ± 52.4^bef^	129.2 ± 43.8^cf^	196.1 ± 66.2^bc^	*P* < *0.001*
	Group B: 21–40 yr (*n* = 20)	116.5 ± 40.1^bh^	149.3 ± 50.1^ch^	229.9 ± 77.2^bch^	*P* < *0.001*
	Group C: 41–60 yr (*n* = 20)	90.2 ± 49.2^bei^	115.6 ± 86.0^ci^	209.2 ± 108.3^bci^	*P* < *0.001*
	Group D: 61–80 yr (*n* = 20)	51.9 ± 31.5^bfhi^	65.4 ± 30.7^cfhi^	154.9 ± 66.3^bchi^	*P* < *0.001*
	*P-value*	*P* < *0.001*	*P* < *0.001*	*P* = *0.036* < *0.05*	*/*
Puncture Efficacy Ratios (%)	Total (*n* = 80)	88.4 ± 13.2^ab^	71.7 ± 18.5^ac^	49.1 ± 17.2^bc^	*P* < *0.001*
	Group A: 10–20 yr (*n* = 20)	87.2 ± 14.2^b^	75.8 ± 19.8^c^	51.8 ± 20.6^bc^	*P* < *0.001*
	Group B: 21–40 yr (*n* = 20)	91.6 ± 10.5^ab^	76.2 ± 17.4^ac^	53.3 ± 12.4^bc^	*P* < *0.001*
	Group C: 41–60 yr (*n* = 20)	90.3 ± 10.6 ^ab^	70.7 ± 16.1^ac^	45.5 ± 14.8^bc^	*P* < *0.001*
	Group D: 61–80 yr (*n* = 20)	84.1 ± 16.6^ab^	65.5 ± 20.5^ac^	46.5 ± 17.7^bc^	*P* < *0.001*
	*P-value*	*P* = *0.261* > *0.05*	*P* = *0.221* > *0.05*	*P* = *0.340* > *0.05*	*/*
Nerve Injury Risk Ratios (%)	Total (*n* = 80)	1.2 ± 3.7^ab^	7.7 ± 9.9^ac^	17.6 ± 13.8^bc^	*P* < *0.01*
	Group A: 10–20 yr (*n* = 20)	1.8 ± 3.1^ab^	8.5 ± 9.8^ac^	14.9 ± 11.9^bc^	*P* < *0.01*
	Group B: 21–40 yr (*n* = 20)	0.7 ± 2.0^ab^	7.8 ± 11.3^ac^	18.4 ± 14.7^bc^	*P* < *0.01*
	Group C: 41–60 yr (*n* = 20)	0.7 ± 2.2^ab^	7.2 ± 9.4^ac^	19.8 ± 12.1^bc^	*P* < *0.01*
	Group D: 61–80 yr (*n* = 20)	1.9 ± 6.2^b^	7.6 ± 9.9^c^	16.6 ± 14.4^bc^	*P* < *0.01*
	*P-value*	*P* = *0.627* > *0.05*	*P* = *0.985* > *0.05*	*P* = *0.674* > *0.05*	*/*

*P* < 0.05 indicates statistical significance

a. Significant difference between L3-4 and L4-5 within the same age group

b. Significant difference between L3-4 and L5-S1 within the same age group

c. Significant difference between L4-5 and L5-S1 within the same age group

d. Significant difference between Group A and Group B for the same segment

e. Significant difference between Group A and Group C for the same segment

f. Significant difference between Group A and Group D for the same segment

g. Significant difference between Group B and Group C for the same segment

h. Significant difference between Group B and Group D for the same segment

i. Significant difference between Group C and Group D for the same segment

### Areas of traversing nerve root (ATNR) of puncture segments

Across all age groups, the ATNR of L5-S1 markedly exceeded L3-4 and L4-5 (*P* < 0.05). In the overall group and group B, L4-5 was greater than L3-4 (*P* < 0.05). Comparisons within subgroups showed a significantly larger L4-5 ATNR versus L3-4 only in group B (21–40 years) (*P* < 0.05), indicating distinct variance during this period.

The ATNR of L5-S1 markedly exceeded L3-4 and L4-5 in all age subgroups (*P* < 0.05). A statistically insignificant decreasing ATNR trend after age 40 was observed. No significant ATNR differences existed among segments within each age group (*P* > 0.05), implying minimal changes with aging.

The ATNR increased progressively from L3-4 to L5-S1, indicating the highest risks of nerve root injuries during L5-S1 puncture. In the overall group, the ATNR of L5-S1 significantly exceeded L3-4 and L4-5 (*P* < 0.05), and L4-5 was greater than L3-4 (*P* < 0.05) (Table 2 and Fig. 5).

### Area of lumbar inter-laminar space (ALILS) of puncture segments

Across all age groups and overall, the ALILS of L5-S1 markedly exceeded L3-4 and L4-5 (*P* < 0.05). No significant differences existed between L3-4 and L4-5 (*P* > 0.05).

Comparisons within subgroups revealed no significant ALILS differences between L3-4 and L4-5 in any age group (*P* > 0.05), indicating minimal variance between L3-4 and L4-5 across age subgroups.

In all subgroups, the ALILS of L5-S1 was markedly greater than L3-4 and L4-5 (*P* < 0.05). A decreasing ALILS trend after age 40 was observed.

Within L3-4, the ALILS of groups A and B were comparable and greater than groups C and D (*P* < 0.05), suggesting relative stability between 10–40 years before a gradual decrease.

Within L4-5 and L5-S1, groups A, B, and C had similar ALILS which exceeded group D (*P* < 0.05), indicating a noticeable ALILS decline from 61–80 years at these segments.

In all age groups and overall, the ALILS of L5-S1 significantly exceeded L3-4 and L4-5 (*P* < 0.05) (Table 2 and Fig. 5).

### Puncture efficacy ratios (ALDS/ALILS) of lumbar segments

In the overall group, the puncture efficacy ratio was lowest at L5-S1, followed by L4-5, and highest at L3-4 (*P* < 0.05).

In group A (ages 10–20 years), the L5-S1 ratio was significantly lower than L3-4 and L4-5 (*P* < 0.05), indicating the lowest efficacy in this subgroup.

In groups B, C, D, and overall (ages 21–80 years), the L5-S1 ratio was markedly lower than L4-5, which was lower than L3-4 (*P* < 0.05), showing the lowest efficacy at L5-S1, followed by L4-5 and highest at L3-4.

The efficacy ratios remained relatively stable across aging. No significant differences existed among segments within each age group (*P* > 0.05) (Table 2 and Fig. 5).

However, the efficacy ratio showed a decreasing trend from L3-4 to L5-S1, indicating that given successful insertion into the interlaminar space, the probability of effective dural puncture was highest at L3-4, followed by L4-5 and lowest at L5-S1.

### Nerve injury risk ratios (ATNR/ALILS) of lumbar segments

Across all age groups, the nerve injury risk ratio was highest at L5-S1, followed by L4-5, and lowest at L3-4 (*P* < 0.05).

In groups A, B, and C (ages 10–60 years), the L5-S1 ratio markedly exceeded L3-4 and L4-5, while L4-5 exceeded L3-4 (*P* < 0.05), indicating increased nerve injury risks with descending puncture segments.

In group D (ages 61–80 years), L5-S1 exceeded L3-4 and L4-5 (*P* < 0.05), while L3-4 and L4-5 were comparable (*P* > 0.05). No significant differences existed among segments within each age group (*P* > 0.05) (Table 2 and Fig. 5).

However, the risk ratio showed an increasing trend from L3-4 to L5-S1, signifying the highest injury probability at L5-S1, followed by L4-5, and lowest at L3-4 given insertion into the interlaminar space.

## Discussion

Lumbar puncture segment selection should be tailored to individual patient conditions, symptoms, and clinical requirements [7]. The main described sites are L2-3, L3-4, L4-5, and L5-S1, with L3-4, L4-5, and L5-S1 being the most commonly used [11]. These interspaces have relatively large windows, enabling easier needle insertion and cerebrospinal fluid sampling with minimal patient injury. The locations of the spinal cord and nerve roots must also be considered to prevent damage [12].

Population aging warrants important consideration in modern medicine. Spinal degeneration and stenosis in the elderly may affect conventional puncture site selection. Our study provides quantitative anatomical evidence supporting L5-S1 as an alternative segment for older patients. Population aging may lead to broader L5-S1 puncture applications. Clinicians should recognize the significant impact of demographic changes on neurological procedure selection.

L1-2 and L2-3 are not first-line options due to spinal cord risks. In tethered cord syndrome, even lower segments may be needed given the low conus position [13]. Sacral dimples and other markers indicating a tethered cord warrant imaging prior to puncture [14].

This study utilized 3D CT reconstruction to obtain precise lumbar anatomy measurements, compared quantitative features of each segment, provided a rationale for optimized selection, and designed innovative efficacy ratio and risk ratio metrics for objective evaluation [15].

Most current studies use 2D measurements [16], rarely applying 3D techniques for large-scale quantitative analysis. This study employed this novel technology to acquire more accurate anatomical data.

This is the first study proposing the concepts of puncture efficacy and nerve injury risk ratios, providing quantitative evidence supporting individualized, age-optimized lumbar puncture segment selection based on these parameters.

### ALDS trends with aging

Our results showed no significant ALDS differences among the L3-4, L4-5, and L5-S1 puncture segments across all age groups (*P* > 0.05), indicating comparable dural exposure. Analyses of subgroups aged 10–20 years, 21–40 years, and 41–60 years also showed no significant ALDS distinctions among the segments (*P* > 0.05), suggesting maintained similarities until age 60.

However, in the 61–80 year cohort, the L5-S1 ALDS (69.4 ± 37.8 mm^2^) markedly exceeded L3-4 (43.6 ± 24.3 mm^2^) and L4-5 (41.6 ± 19.1 mm^2^) (*P* < 0.05), implying superior L5-S1 outcomes in the elderly.

The ALDS remained stable from ages 10–40 years before declining, with the smallest decrease at L5-S1. Thus, L5-S1 may provide an alternative when traditional upper lumbar access becomes difficult for the elderly.

These findings align with age-related degeneration. Disc height loss and stenosis from osteophytes and thickened ligaments can compress the dural sac [17–19]. The later and smaller ALDS decline at L5-S1 relates to its larger native window better accommodating changes before severe narrowing [20].

In summary, L5-S1 may be an alternative when traditional upper lumbar puncture sites become challenging in older patients, explaining potentially improved outcomes. Our quantitative data provides insights into the anatomical basis underlying these clinical observations.

### ATNR differences among segments

Our results showed significant ATNR differences among the L3-4, L4-5, and L5-S1 puncture segments across all age groups (*P* < 0.05). The L5-S1 ATNR was 34.0 ± 27.8 mm^2^, markedly exceeding 9.0 ± 14.0 mm^2^ at L4-5. Meanwhile, the L4-5 ATNR also exceeded 1.1 ± 3.1 mm^2^ at L3-4. Similar trends were seen in subgroups A, B, and C.

In the older cohort D, the L5-S1 ATNR remained significantly higher at 25.9 ± 22.2 mm^2^ versus 5.2 ± 7.6 mm^2^ at L4-5 (*P* < 0.05). The L4-5 and L3-4 difference of 0.6 ± 1.3 mm^2^ was insignificant (*P* > 0.05).

The larger lower lumbar ATNRs may relate to the inferior nerve root exit points from the dural sac, coupled with their wider diameters and smaller traversing angles, predisposing the S1 roots to injury during puncture [21].

The greater ATNRs in lower segments can be attributed to the acute angles and inferior origins of the L5 and S1 nerve roots. Studies show these roots emanate lower and have more oblique courses compared to upper roots [22]. The S1 roots demonstrate particular vulnerability at L5-S1, heightening damage risks [21].

Our quantitative data provides an anatomical basis for the substantially higher incidence of S1 versus other lumbar radiculopathies. The findings underscore the critical importance of extreme caution and optimal trajectory planning during L5-S1 puncture.

### ALILS changes with aging

The interlaminar space area (ALILS) is clinically important, as the osseous structures outside the ALILS provide crucial tactile feedback during lumbar puncture, enabling adjustment of the needle trajectory to enter the ALILS.

Our results showed the L5-S1 ALILS significantly exceeded L3-4 and L4-5 across all age groups (*P* < 0.05), indicating L5-S1 allows the easiest canal access. Overall, the ALILS was 197.6 ± 90.3 mm^2^ at L5-S1, 113.8 ± 65.3 mm^2^ at L4-5, and 93.4 ± 51.0 mm^2^ at L3-4, with L5-S1 exceeding L3-4 two-fold.

From ages 10–40 years, the ALILS remained relatively constant. After age 40, the ALILS declined across segments [20]. The consistently larger L5-S1 window was maintained at a greater size for longer, enabling its potential use for complex punctures. The greater L5-S1 ALILS may relate to the lumbosacral junction anatomy and biomechanics [23], with evolutionary enlargement to accommodate spinal-pelvic forces [20]. Our findings align with degeneration primarily affecting L3-4 and L4-5 [24]. Biomechanical stresses likely contribute to earlier upper-level stenosis [25].

The innately wider L5-S1 window better accommodates age-related changes before severe compression [26], allowing its potential use for difficult access when conventional sites narrow [7]. Our quantitative ALILS data provides anatomical support for this technique.

### Puncture efficacy ratio changes with aging

In clinical practice, we frequently encounter scenarios where the needle passes into the epidural space through the interlaminar window (ALILS), yet fails to penetrate the dura to access the subarachnoid space (ALDS). This nuance is often overlooked, and our quantitative analysis of ALILS and ALDS provides evidence-based guidance for optimal puncture segment selection to avoid such dilemmas. For instance, our data showed preferentially selecting L3-4 maximizes the chances of entering ALDS after penetrating ALILS, as it had the highest efficacy ratio. We also summarized the varying characteristics of ALILS and ALDS across lumbar segments, enabling clinicians to choose appropriate intervertebral spaces to increase overall puncture success. When facing ALDS access failure after entering ALILS, we can advise modifications based on our segmental measurements. Overall, delineating these anatomical differences quantitatively supports optimized, patient-tailored puncture techniques.

Our results demonstrated significant efficacy ratio differences among the lumbar segments across all age groups (*P* < 0.05). The highest ratio was at L3-4 (88.4 ± 13.2%), followed by L4-5 (71.7 ± 18.5%), and lowest at L5-S1 (49.1 ± 17.2%).

The efficacy ratios remained relatively stable across aging for each level, indicating the highest dural puncture likelihood at L3-4, followed by L4-5, and lowest at L5-S1 given successful interlaminar insertion [27].

When accessible, L3-4 and L4-5 should be preferred over L5-S1, as their bone provides superior tactile feedback during needle advancement. L5-S1 risks neural complications like hematoma, nerve injury, and disc damage [28].

Despite being statistically insignificant, the declining efficacy ratio order from groups B to D suggests age-related dural compression [29]. Studies also report higher L4-5 iatrogenic disc injury risks [30], potentially causing degeneration and herniation [31].

Ultimately, individual factors determine optimal segment selection, such as disc herniation or stenosis may necessitate alternate levels, unilateral radiculopathy favors a contralateral approach.

The stable efficacy ratios from ages 10–40 years coincide with minimal degenerative changes during this period [32]. The subsequent declining trend after middle age aligns with reduced disc height, hypertrophic facets/laminae, and buckled ligaments [33, 34], preferentially narrowing the dural space [35].

Our data advocates preferential L3-4 and L4-5 puncture when feasible, as L5-S1 risks neural injury and requires subarachnoid space expertise [7]. However, L5-S1 should be considered as an alternative if upper lumbar stenosis necessitates more caudal access [11, 36].

### Nerve injury risk ratio changes with aging

While transient radicular symptoms during lumbar puncture may not be uncommon (13%) [37], more sustained nerve injury appears to be rare [38]. Our results demonstrated significant differences in the nerve injury risk ratios (ATNR/ALILS) among the lumbar segments across all age groups (*P* < 0.05). The highest ratio was at L5-S1 (17.6 ± 13.8%), followed by L4-5 (7.7 ± 9.9%), and lowest at L3-4 (1.2 ± 3.7%). Similar trends were seen in subgroups A, B, and C.

Currently, most LPs are performed at the L3-4 and L4-5 segments, with less frequent use of L5-S1. The lower inherent exposure of traversing nerve roots at L3-4 and L4-5 results in lower injury risks. However, L5-S1 puncture increases proportional nerve root exposure due to the larger interlaminar space, elevating injury risks – a key point of this study.

Furthermore, the effective dural puncture area (ALDS) decreased after age 40 and was markedly reduced at L3-4 and L4-5 in group D patients (ages 61–80 years). In such cases, L5-S1 may need to be used despite lower efficacy and higher injury risks, concerning for clinicians.

In response to calculating injury risks, while post-LP nerve injury is rare, our aim was to provide an additional metric for potential risks. This can help guide decisions between segments, particularly in borderline scenarios.

In the older cohort D, the L5-S1 risk ratio remained markedly higher at 16.6 ± 14.4% versus 7.6 ± 9.9% at L4-5 (*P* < 0.05), while there was no significant difference between L4-5 and L3-4 (1.9 ± 6.2%, *P* > 0.05).

The greater lower lumbar risk ratios may relate to the differing nerve root exit points, diameters, and angles originating from these segments [39], heightening S1 injury risks during attempted L5-S1 puncture.

The rising L3-4 to L5-S1 risk ratios parallel the progressively larger and more horizontal transverse nerve roots at the lower levels [40]. The wide diameters and tethering of S1 roots within the recesses predispose them to damage. Rampersaud et al. showed the S1 roots occupied over half the L5-S1 epidural space based on cadaver measurements [41].

Although the risk ratios were relatively stable across aging, caution is still warranted for caudal punctures. Precise alignment and controlled needle advancement are critical to prevent irreparable neural damage [8]. Ultrasonography can also improve difficult L5-S1 access safety [7].

### Study limitations and future research directions

This study has several limitations.

First, CT imaging was performed in supine rather than flexion position typical for lumbar puncture. Though supine imaging follows standard protocols, flexion positioning would better approximate puncture anatomy. Paired LP and CT imaging in all subjects was not feasible presently due to ethical constraints. Further studies with paired imaging could help validate our methodology.

Second, our cohort was restricted to participants meeting strict criteria. Further studies should evaluate more diverse populations, expanded age ranges, and high-risk groups like obesity and scoliosis to enhance generalizability.

Third, we performed computational measurements without clinical correlation. Future research could integrate anatomical assessments with lumbar puncture outcomes data to validate which parameters predict procedural success.

In this analysis, we isolated the effect of needle trajectory angle by examining the efficacy ratio at the optimal puncture angle, without considering the needle entry point margin of error and skin-dura distance. This is a limitation, as incorporating more variables would enable a more comprehensive model. In future studies, will incorporate entry point precision and skin-to-dura distances to provide a robust simulation of real-world puncture scenarios. This phased approach will enhance the clinical applicability of our modeling methodology.

Despite limitations, our study establishes a novel quantitative foundation for informing age-specific, patient-tailored lumbar puncture techniques balancing safety and efficacy. Advances in digital spine modeling and analytics will continue enhancing evidence-based procedural guidance.

## Conclusions

The present study demonstrates that the L3-4 intervertebral space confers optimal puncture efficacy and lowest nerve injury risk ratios. Hence, L3-4 is concluded as the preferred first-choice lumbar puncture segment for patients aged 10–60 years, enabling optimized safety and effectiveness. When L3-4 and L4-5 interspaces become stenotic, L5-S1 can serve as an alternative option in elderly patients over 60 years.

This is the first study utilizing 3D imaging to obtain quantitative parameters of lumbar interspaces including effective dural sac area, traversing nerve root area, and interlaminar space area. The novel concepts of puncture efficacy ratio and nerve injury risk ratio are also introduced. The findings provide quantitative imaging evidence supporting optimized and individualized lumbar puncture segment selection across different age groups.

## Data Availability

All data generated or analyzed during this study are available from the authors upon reasonable request.
